# Hailey-Hailey disease successfully treated with tralokinumab and literature review of successful treatment with dupilumab

**DOI:** 10.1016/j.jdcr.2024.07.008

**Published:** 2024-07-27

**Authors:** Kareena S. Garg, Jonathan Silverberg, Leonardo Tjahjono

**Affiliations:** a Georgetown University School of Medicine, Washington, District of Columbia; b Department of Dermatology, The George Washington University School of Medicine and Health Sciences, Washington, District of Columbia; c Pinnacle Dermatology, Woodbridge, Virginia

**Keywords:** benign familial pemphigus, blistering disease, genodermatosis, Hailey-Hailey disease, interleukin 13, tralokinumab

## Introduction

Familial benign chronic pemphigus, also known as Hailey-Hailey disease (HHD), is a rare autosomal dominant genodermatosis that presents as chronic, painful, erythematous, erosive plaques, and fissures. Patients with HHD often suffer from comorbid impacts on psychosocial health and quality of life. HHD is due to a mutation in the *ATP2C1* gene on chromosome 3, leading to defective Golgi apparatus calcium homeostasis, disruption in keratinocyte adhesion, and consequent development of intraepidermal acantholysis. Traditional medical treatment options for HHD are limited and have unreliable efficacy; these include topical steroids and calcineurin inhibitors, topical and systemic antibiotics, oral retinoids, and opioid modulators.^1^

Tralokinumab is a novel interleukin 13 (IL-13) inhibitor. It is Food and Drug Administration–approved for atopic dermatitis in patients 12 years and older. We report a case of HHD refractory to conventional treatments, successfully treated with tralokinumab samples provided by manufacturer.

## Case report

**Fig 1:**
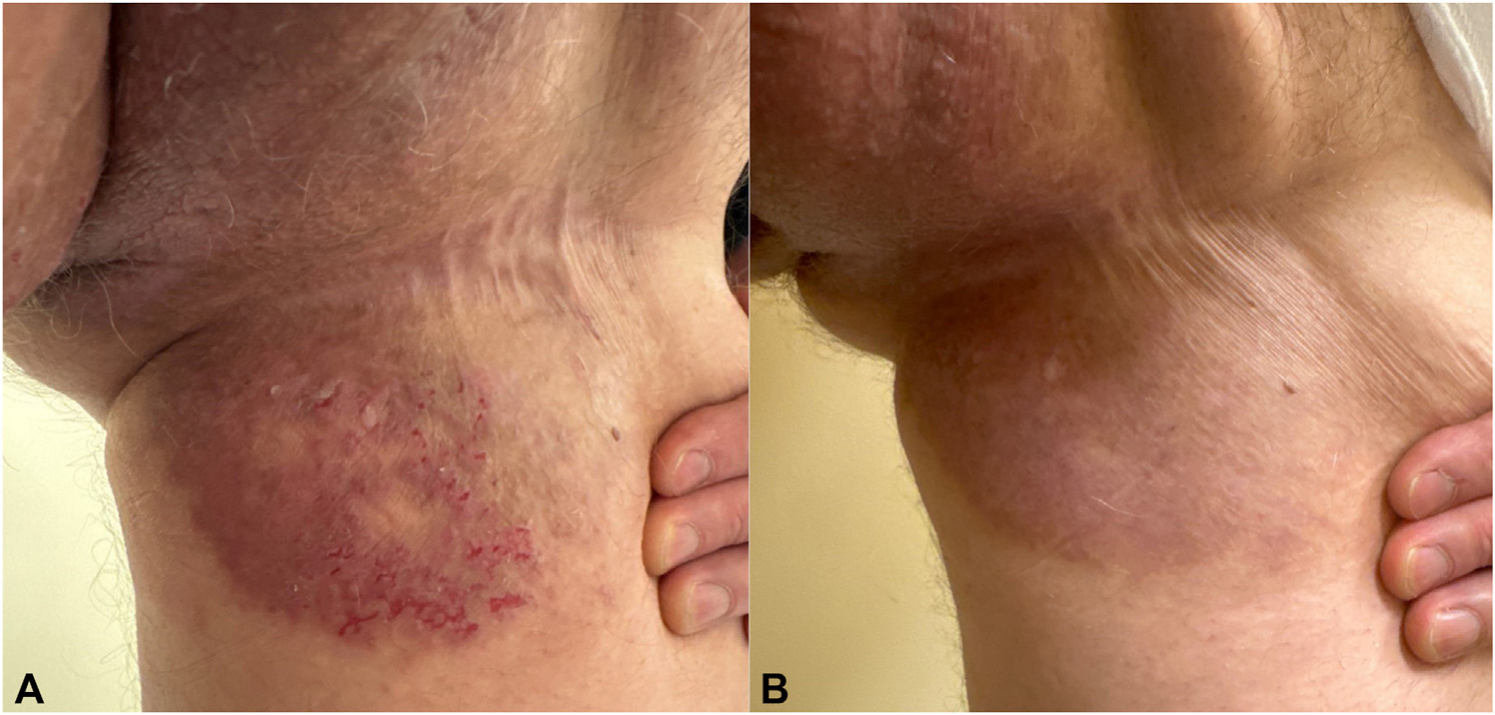
Hailey-Hailey disease of the left thigh; resolution of painful, erosive, erythematous plaque (**A**) after 6 weeks of tralokinumab (**B**).

**Fig 2:**
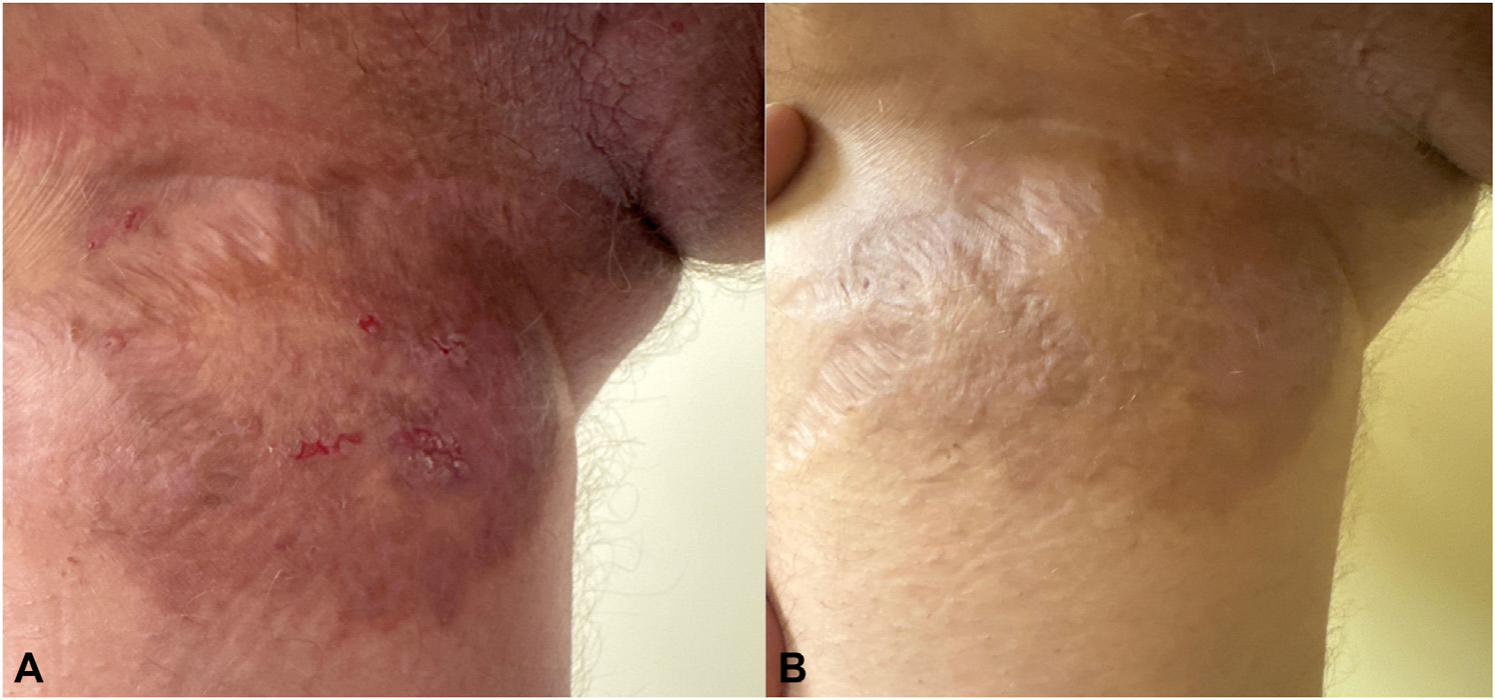
Hailey-Hailey disease of the right thigh; resolution of painful, erosive erythematous plaque (**A**) after 6 weeks of tralokinumab, except for cutaneous atrophy in the setting of long-term topical corticosteroids use (**B**).

A 58-year-old man with past medical history of hypertension, hyperlipidemia, and a 20-year history of chronic HHD presented to clinic as a referral with a severe flare of painful erythematous, eroded pink plaques on his inner thighs. He does not have any known family history of HHD. Multiple biopsies from external facilities showed epidermal hyperplasia, parakeratosis, and diffused epidermal acantholysis with negative direct immunofluorescence, supportive for HHD. The disease was recalcitrant to long-term trial of topical antibiotics, systemic antibiotics, topical corticosteroids, and systemic naltrexone for 20 years with persistent pain and no significant improvement in cutaneous presentation. After receiving tralokinumab loading dose of subcutaneous 600 mg and subsequent 300 mg every 2 weeks, his pain symptoms started to improve after the first 300-mg dose. He was then able to discontinue topical corticosteroids and topical antibiotics. The erythematous, eroded plaques resolved on his 6-week follow-up (Figs 1 and 2). He was able to maintain remission on 3-month follow-up with subcutaneous 150 mg of tralokinumab every 4 weeks without any adverse effect throughout duration of his treatment. The patient reported that he was able to perform physical exercise for the first time in decades.

## Discussion

Tralokinumab, an IL-13 inhibitor, has a similar mechanism of action as dupilumab by inhibiting the effect of downstream cytokines produced by T-helper 2 cell, which subsequently dampens stimulation of eosinophils and basophils. Dupilumab has been reported to improve HHD in the literature, which we summarized in Table I^2^, ^3^, ^4^, ^5^, ^6^ While the mechanism is unclear, a possible explanation of tralokinumab’s efficacy is that IL-13 inhibition increases influx of calcium into keratinocytes, which is critical for normal keratinocytes differentiations and cellular adhesion; this likely negates abnormal intracellular calcium signaling caused by HHD genetic defect.^7^ Tralokinumab and IL-13 inhibition also attenuate C-C chemokine receptor type 3 and eotaxin-3 interactions downstream effect, which attract eosinophils and basophils; both cells inhibit the release of intracellular calcium.^8^ The attenuation likely promotes influx of calcium into keratinocytes, promoting normal keratinocytes differentiations and cellular adhesion.

**Table I:** Summarized Hailey-Hailey disease treated with dupilumab in the literature

Gender, age	Prior treatments	Outcome
F, 50s	A, I, CsA, Ah, OABX, TCS, Pr, Et, TCS, LDN, SAZ	Sustained improvement after 21 mo
M, 50s	A, TCS, LDN, OG	Sustained improvement after 25 mo, reflared when treatment was interrupted
M, 70s	Botox, ICS	Sustained improvement after 17 mo
F, 56	A, Ah, AP, CsA, SAZ, DS, HCQ, LDN, OABX, MTX, oxybutynin, Pr, sulfone, TCS, TCI	Significant improvement after 2 mo and sustained for 14 mo
M, 52	A, AP, CO2 laser, OABX, LDN, MMF, oxybutynin, Pr, TCS	No improvement after 13 mo of treatment
F, 59	A, Ah, AP, DS, LDN, OABX, Pr, TCS	Significant improvement after 5 mo and sustained for 16 mo
F, 22	TCS, Ah, CsA	Significant improvement after 4 mo
F, 53	TCI, OABX, topical erythromycin, ICS, TCS, AP	Significant improvement after 2 wk, with further improvement with concomitant use of topical ruxolitinib 1.5% cream

*A*, Acitretin; *Ah*, antihistamine; *AP*, apremilast; *CsA*, cyclosporine; *DS*, dapsone; *Et*, etanercept; *HCQ*, hydroxycholoroquine; *I*, isotretinoin; *ICS*, intralesional corticosteroids; *LDN*, low dose naltrexone; *MMF*, mycophenolate mofetil; *MTX*, methotreaxate; *OABX*, oral antibiotics; *OG*, oral glycopyrrolate; *Pr*, prednisone; *SAZ*, sulfadiazine; *TCI*, topical calcineurin inhibitor; *TCS*, topical corticosteroids.

To our knowledge, this is the first report of tralokinumab as a treatment option for HHD. While promising, further controlled studies are needed to evaluate tralokinumab as a treatment option for recalcitrant HHD.

## Conflicts of interest

Dr Tjahjono has served as a consultant for Bristol Myers Squibb. Dr Silverberg is an advisor, speaker, or consultant for AbbVie, Asana Biosciences, Dermavant, Galderma, GlaxoSmithKline, Glenmark, Kiniksa, LEO Pharma, Lilly, Menlo Therapeutics, Novartis, Pfizer, Realm Pharma, and Regeneron-Sanofi; and also a researcher for GlaxoSmithKline. Author Garg has no conflicts of interest to declare.
